# The AWARE (AI Use, Why, Attachment, Reality and Risk, and Effect on Functioning) Framework for Assessing Patients’ Use of AI in Mental Health Care

**DOI:** 10.2196/108766

**Published:** 2026-09-16

**Authors:** Alexandre Hudon, Venkat Bhat, John-Jose Nunez, Vincent Paquin, Amylie Malouin-Lachance, François Lespérance

**Affiliations:** 1 Department of Psychiatry and Addictology Faculty of Medicine Université de Montréal Montreal, QC Canada; 2 Forensic psychiatry services Department of Psychiatry Institut universitaire en santé mentale de Montréal Montreal, QC Canada; 3 Centre de recherche de l'Institut universitaire en santé mentale de Montréal Montreal, QC Canada; 4 Department of Psychiatry Institut national de psychiatrie légale Philippe-Pinel Montreal, QC Canada; 5 Centre de recherche en pédagogie de la santé Université de Montréal Montreal, QC Canada; 6 Department of Psychiatry University of Toronto Toronto, ON Canada; 7 Department of Psychiatry AI for Mental Health St. Michael's Hospital Toronto, ON Canada; 8 Department of Psychiatry University of British Columbia Vancouver, BC Canada; 9 Department of Computer Science University of British Columbia Vancouver, BC Canada; 10 Department of Psychiatry McGill University Montreal, QC Canada; 11 Lady Davis Institute for Medical Research Jewish General Hospital Montreal, QC Canada; 12 Department of Psychiatry Faculté de médecine Université Laval Québec, QC Canada; 13 Département de psychiatrie Centre Hospitalier de l’Université de Montréal Montreal, QC Canada; 14 Centre de recherche du Centre hospitalier de l’Université de Montréal Montreal, QC Canada

**Keywords:** artificial intelligence, AI, conversational artificial intelligence, large language models, ChatGPT, psychiatry, mental health, psychiatric interview, clinical assessment, medical education, digital mental health

## Abstract

Conversational AI has rapidly become integrated into everyday life, with many individuals using large language model–based systems, such as ChatGPT, Gemini, and Claude, for education, productivity, health information, decision-making, emotional support, and companionship. As patients increasingly incorporate conversational AI into their cognitive, emotional, and social lives, these interactions may influence coping strategies, treatment engagement, clinical decision-making, and mental health outcomes. However, although medical education has increasingly emphasized AI literacy and the responsible use of generative AI by clinicians, comparatively little attention has been given to preparing health care professionals to assess patients’ use of conversational AI during routine clinical encounters. This Viewpoint introduces the AWARE (AI use, why, attachment, reality and risk, and effect on functioning) framework, a practical educational framework designed to help mental health professionals systematically assess patients’ use of conversational AI. Rather than functioning as a diagnostic instrument or psychometric scale, AWARE provides a structured approach to psychiatric interviewing across 5 clinically relevant domains: AI use, why, attachment, reality and risk, and effect on functioning. Together, these domains guide clinicians in exploring patterns of AI use, the motivations underlying engagement, the emotional significance of patient-AI interactions, potential influences on reality testing and clinical risk, and the overall impact of AI on psychological well-being, daily functioning, relationships, and recovery. The framework is intended to complement existing psychiatric interviewing practices by supporting comprehensive history taking, clinical reasoning, risk assessment, documentation, and learner education. We discuss the rationale for routinely asking patients about AI use, review emerging evidence regarding both the potential benefits and risks of conversational AI, and describe how AWARE may be incorporated into undergraduate, postgraduate, and continuing professional education through simulation, objective structured clinical examinations, workplace-based assessment, and clinical supervision. We also outline priorities for future research, including validation, implementation, educational evaluation, and cross-cultural adaptation. As conversational AI becomes increasingly embedded within patients’ daily lives, clinicians require practical approaches to understanding its role in mental health and health care. The AWARE framework offers a structured educational starting point for integrating assessment of patient-AI interactions into routine psychiatric interviewing while supporting patient-centered, evidence-informed clinical practice and health professions education.

## Introduction

### The Rapid Integration of AI Into Everyday Life

The public release of large language model (LLM)–based systems, such as ChatGPT (OpenAI), Gemini (Alphabet Inc), and Claude (Anthropic PBC), has changed how people interact with AI. Unlike earlier narrowly defined applications, conversational AI supports natural language dialogue across education, problem-solving, decision-making, and personal concerns and has rapidly become integrated into everyday life [1,2].

Conversational AI nevertheless encompasses heterogeneous technologies that should not be treated as interchangeable. General-purpose LLM assistants are designed for broad conversational use, whereas purpose-built mental health agents such as Woebot (Woebot Labs Inc) and Wysa (Touchkin Eservices Pvt Ltd) deliver structured psychological or well-being interventions, and companion chatbots such as Replika (Luca Inc) emphasize sustained relational interaction. Evidence from 1 category should therefore not be assumed to generalize to another. In this Viewpoint, we distinguish these technologies when interpreting evidence while using “conversational AI” as an umbrella term for systems that interact with users through dialogue.

Use of conversational AI has also expanded beyond productivity and education [1,3] to emotional support, companionship, self-reflection, health information, and personal decision-making. In a survey of 1006 university students using Replika, participants described the companion chatbot in overlapping roles, including friend, therapist, and intellectual mirror, and a small subgroup reported that interactions had interrupted suicidal thinking [4]. These findings are specific to a companion-oriented system and should not be generalized to general-purpose LLMs. Surveys of ChatGPT users similarly indicate growing use for health-related questions and health care decision-making, with perceived trustworthiness influencing reliance on AI-generated recommendations [5].

These patterns are clinically relevant because patients may discuss symptoms with AI before seeing a clinician, seek emotional support during distress, or incorporate AI-generated information into their understanding of mental health and treatment. Understanding how patients use and interpret these systems is therefore becoming increasingly relevant to contemporary psychiatric assessment.

### AI Represents a Fundamentally Different Digital Exposure

The adoption of conversational AI represents a fundamentally new type of digital exposure. Earlier technologies, including search engines and social media, primarily provided access to information or facilitated interactions among people. Although these technologies can influence beliefs, emotions, and behavior, they do not themselves function as conversational partners. In contrast, LLM-based conversational agents engage users directly in dialogue, generating personalized responses that adapt to language, preferences, and conversational context. Their ability to sustain coherent, context-sensitive exchanges creates a qualitatively different interaction in which AI becomes an active conversational participant.

Several features distinguish conversational AI from earlier digital technologies. Rather than relying on keywords or predefined commands, these systems support natural language interaction and iterative multiturn dialogue in which exchanges evolve dynamically. Their personalization and continuous availability further make interactions more intuitive and responsive. They can also generate responses that users may experience as empathic, supportive, validating, or emotionally responsive [6-9]. Such perceived relational qualities should not be equated with human empathy or reciprocal interpersonal understanding; rather, they reflect the capacity of conversational systems to generate context-sensitive language that can be experienced as socially and emotionally meaningful.

Evidence for perceived therapeutic alliance comes primarily from purpose-built mental health conversational agents rather than contemporary general-purpose LLMs. Users of Wysa, for example, reported working alliance scores comparable with those reported in previous studies of human-delivered and internet-based psychotherapy [6]. Systematic reviews similarly suggest that relational factors may contribute to engagement and user experience, although the included technologies are heterogeneous and findings should not be extrapolated directly to general-purpose LLM systems [10].

Experimental studies further suggest that meaningful relational processes can emerge when interactions are perceived as chatbot- or AI-mediated. Ho et al [7] found broadly similar effects of emotional self-disclosure when participants believed they were conversing with a chatbot or another person. Barger [8] found that participants could report a working alliance with a coach they believed to be AI; however, the study used a Wizard-of-Oz design in which responses were actually generated by professional human coaches. This finding therefore informs the role of perceived agent identity rather than demonstrating that an autonomous AI system can independently establish such an alliance. In another experimental study, chatbot emotional disclosure was associated with greater perceived intimacy, user satisfaction, and intention to reuse the service [9]. Together, these findings indicate that relational experiences can emerge in interactions perceived as AI-mediated, while underscoring that evidence from experimental and purpose-built systems should not be generalized directly to contemporary general-purpose LLMs.

From a psychiatric perspective, this matters because conversational AI can simulate interpersonal behaviors traditionally associated with human relationships, including empathy, validation, encouragement, and reassurance. Although these interactions should not be assumed to involve reciprocal human-like understanding, users may nevertheless experience them as socially or emotionally meaningful, with implications for beliefs, emotions, coping, and decision-making. Conversational AI may therefore be understood not simply as another digital technology but as a novel relational environment. Earlier digital platforms have long supported parasocial and other psychologically meaningful interactions, but conversational AI introduces a more direct, adaptive, and personalized form of engagement whose effects on psychological functioning are only beginning to be understood.

### Emerging Psychiatric Implications

Conversational AI presents both opportunities and challenges for mental health care. Purpose-built conversational agents can provide psychoeducation, cognitive behavioral therapy (CBT)–based exercises, symptom monitoring, and coping strategies in accessible and scalable formats [10-14]. Evaluations of systems such as Woebot and Wysa suggest potential benefits for depressive and anxiety symptoms, acceptability, and engagement [11-13], supporting their possible role as adjuncts to care, particularly when access to professional services is limited.

The same features that make conversational AI accessible and engaging may also create clinically relevant risks. These systems can sustain personalized, emotionally meaningful interactions that encourage repeated disclosure and may become sources of reassurance, companionship, validation, or therapeutic guidance [6-10]. Case reports describe reinforcement of delusional beliefs and unsafe health-related decision-making [15,16], while other studies raise concerns about emotional reliance, dependency, and displacement of human relationships [4,8,17]. General-purpose LLMs also vary in their responses to suicidal crises, with some responses diverging from established prevention principles [18,19]. Evidence regarding violence-related interactions remains limited.

The evidence base remains preliminary and heterogeneous, consisting largely of feasibility studies, observational research, cross-sectional surveys, and case reports [10,20]. Conversational AI should therefore not be regarded as inherently beneficial or harmful. Rather, its clinical significance is likely to depend on the patient’s motivations, pattern of use, vulnerabilities, social context, and the ways AI interacts with symptoms, coping, interpersonal functioning, and treatment engagement.

### The Educational Gap

Although medical education has rapidly embraced generative AI as a tool for teaching, learning, and clinical decision support, considerably less attention has been devoted to preparing clinicians to assess patients’ own AI use during routine clinical encounters [21,22]. Existing educational initiatives primarily focus on AI literacy, ethical use of AI, prompt engineering, and the incorporation of AI into medical curricula rather than its implications for psychiatric assessment or patient care [21,22]. Consequently, clinicians may be well prepared to use AI themselves while remaining unfamiliar with how patients incorporate conversational AI into their emotional, cognitive, and social lives.

This educational gap is particularly evident in psychiatric interviewing. Existing psychiatric assessment frameworks and clinical guidance increasingly encourage clinicians to explore domains such as substance use, digital behaviors, internet and social media use, interpersonal relationships, and coping strategies because each may influence diagnosis, risk assessment, and treatment planning [20,23]. However, these frameworks were largely developed before the widespread adoption of conversational agents and provide limited practical guidance for assessing patients’ interactions with generative AI. Although emerging publications have begun to address this issue, no widely adopted or standardized approach has yet been incorporated into routine psychiatric assessment. Consequently, clinicians may overlook clinically relevant information regarding the frequency, purpose, emotional significance, and influence of AI interactions despite their growing role in patients’ everyday lives.

### Why Clinicians Should Ask About AI

As conversational AI becomes integrated into everyday life, patients may use it not only for information but also for emotional support, symptom interpretation, and health-related decision-making. This creates an important parallel with clinicians, who increasingly encounter generative AI for learning, information synthesis, and clinical decision support [5,21,22]. Patients may similarly use these systems to interpret symptoms, consider possible diagnoses, prepare for clinical encounters, or weigh treatment decisions. Clinicians therefore need practical ways to explore not simply whether patients use AI, but how AI-generated information and interactions have entered their understanding of health, relationships, and care.

### Objective

The objective of this Viewpoint is to operationalize emerging recommendations that clinicians assess patients’ use of conversational AI by introducing the AWARE (AI use, why, attachment, reality and risk, and effect on functioning) framework, a practical educational tool that organizes this assessment into 5 clinically relevant domains and provides structured interview questions for their exploration. AWARE organizes this inquiry across 5 clinically relevant domains: *AI use*, *why*, *attachment*, *reality and risk*, and *effect on functioning*. Although primarily intended for education and training, the framework may also serve as a flexible interviewing guide when a patient’s AI use is disclosed or judged clinically relevant. It is not intended as a screening instrument, diagnostic tool, risk-prediction instrument, or standardized clinical protocol. Rather than assuming that AI use is either beneficial or harmful, AWARE encourages clinicians and learners to consider how conversational AI may interact with an individual patient’s mental health, functioning, relationships, and care.

Although the acronym AWARE has previously been used by Lombardo et al [24] for a framework addressing clinical decision-making in crisis resolution and home treatment teams, the present framework is conceptually and operationally distinct and focuses specifically on assessing patients’ interactions with conversational AI. Recent work has begun to explicitly recommend that clinicians ask patients about AI use and evaluate potentially risky patterns of engagement [25]. AWARE builds on these recommendations by providing a mnemonic structure and concrete interview questions that operationalize such assessment in clinical practice.

### AI Is Becoming Part of Patients’ Social Environments

Patients increasingly use conversational AI for purposes that extend beyond information retrieval. Depending on individual needs and circumstances, AI may function as a teacher for learning new concepts, a coach for improving performance or productivity, a source of health information, a decision aid for navigating complex choices, or a conversational partner for emotional support and self-reflection [1-5]. Some individuals describe AI as a confidant with whom they share experiences they would hesitate to disclose to others. In contrast, others report using conversational agents in ways that resemble psychotherapy or companionship [4-10].

The relational affordances of conversational systems vary substantially by design. Companion agents may be optimized for sustained relational engagement, purpose-built mental health chatbots may structure interactions around therapeutic or well-being content, and contemporary general-purpose LLMs can generate highly personalized, context-sensitive dialogue across unrestricted topics. Despite these technological differences, users may experience interactions with each category as emotionally meaningful. The strength, nature, and clinical implications of these experiences should therefore be evaluated at the level of the specific system and pattern of use rather than generalized across conversational AI technologies.

From a psychiatric perspective, AI should therefore be viewed not only as a technology but also as a novel relational environment that may shape patients’ thoughts, emotions, and behaviors. Understanding how an individual patient incorporates AI into daily life provides important contextual information that may not otherwise emerge during routine psychiatric assessment.

### AI May Influence Clinical Presentation

Conversational AI may interact with existing psychiatric symptoms and vulnerabilities in both adaptive and maladaptive ways. It may provide psychoeducation, coping support, or emotional expression, particularly where access to care is limited [10-14]. Conversely, emerging reports suggest that AI may become involved in reassurance seeking, social substitution, delusional belief systems, treatment decisions, or crisis-related interactions [4,15,17-19]. These observations should not be interpreted as establishing AI as an independent cause of psychopathology. Rather, AI use represents a contextual factor whose clinical significance depends on the patient’s vulnerabilities, motivations, social circumstances, and pattern of use.

### AI Use Should Be Considered During Psychiatric Assessment

Psychiatric interviewing has continually evolved to reflect changes in society and technology. Clinicians now routinely ask about substance use, internet behaviors, social media use, sleep, sexual health, and other lifestyle factors because these domains are important for patients and can guide diagnosis, risk assessment, and treatment planning. These questions are not asked because they presume a pathological behavior, but because they help clinicians understand patients within the broader context of their lives.

Conversational AI increasingly warrants similar consideration. A patient who occasionally uses ChatGPT to summarize lecture notes presents different clinical considerations than someone who spends several hours each day seeking emotional reassurance from an AI companion or who incorporates AI-generated responses into fixed beliefs about themselves or the world. Without asking about AI use, clinicians may overlook important influences on emotional well-being, interpersonal functioning, treatment adherence, and clinical risk.

Routine assessment of AI use should therefore be viewed as an extension of comprehensive psychiatric history taking rather than the creation of an entirely new assessment domain. The objective is neither to pathologize AI use nor to assume that it is beneficial, but rather to understand how conversational AI influences an individual patient’s psychological functioning and clinical care. As with other components of the psychiatric interview, the depth of AI-related assessment should be guided by clinical relevance, presenting concerns, and available interview time rather than applied uniformly to every patient. To facilitate this process, we propose the AWARE framework as a flexible, structured approach that can be incorporated into routine mental health encounters when clinically indicated.

### Conceptual Development of the AWARE Framework

AWARE was developed as an author-proposed conceptual interviewing framework rather than through a formal qualitative, Delphi, or psychometric development process. The 5 domains were organized through a narrative synthesis of the clinical issues identified in the literature reviewed above and according to their distinct functions within psychiatric interviewing. *AI use* establishes the nature and extent of exposure; *why* examines the motivations and needs underlying use; *attachment* addresses the relational and emotional significance of interactions with conversational AI; *reality and risk* examines whether these interactions influence beliefs, judgment, treatment decisions, or safety; and *effect on functioning* evaluates their overall consequences for daily life, relationships, treatment, and recovery.

The 5 AWARE domains are intended as mnemonic domains of inquiry rather than mutually exclusive psychological constructs. Some overlap is therefore expected. For example, motivation for AI use may relate to attachment, trust may be relevant to both relational significance and appraisal of AI-generated information, and functional consequences may arise from patterns identified in several earlier domains. This redundancy is intentional insofar as AWARE is designed to support clinical interviewing rather than construct measurement. The framework should therefore not be interpreted as proposing 5 orthogonal or psychometrically distinct dimensions of patient-AI interaction.

Attachment was retained as a distinct domain because an emotionally meaningful relationship with conversational AI may be clinically relevant even in the absence of impaired reality testing, overt risk, or functional deterioration. Conversely, reality testing and risk were grouped within a single domain for clinical parsimony because both represent situations in which AI-generated interactions may warrant escalation from descriptive history taking to established psychiatric assessment of judgment, safety, and clinical risk. Their combination does not imply that impaired reality testing and risk are equivalent or necessarily co-occur.

Accordingly, the present 5-domain structure should be understood as a theoretically and clinically informed heuristic proposed for further evaluation, rather than as an empirically established taxonomy of patient-AI interactions. Formal content-validation work involving clinicians, patients, and other relevant stakeholders will be required to determine whether these domains are sufficiently comprehensive, distinct, and clinically useful.

## The AWARE Framework

### Overview

The AWARE framework is an author-proposed conceptual interviewing framework and educational tool designed to help mental health professionals systematically explore patients’ interactions with conversational AI during clinical encounters. Rather than determining whether AI use is inherently beneficial or harmful, AWARE guides clinicians through 5 domains that together characterize the patient’s pattern of use, motivations, emotional relationship with AI, potential safety concerns, and overall impact on functioning.

The framework is intended to complement (not replace) existing psychiatric interviewing techniques by providing a practical structure for exploring an increasingly common aspect of patients’ digital lives. Although the domains are presented sequentially, clinicians may adapt their order according to the clinical context. An overview of the framework is shown in Figure 1.

**Figure 1:**
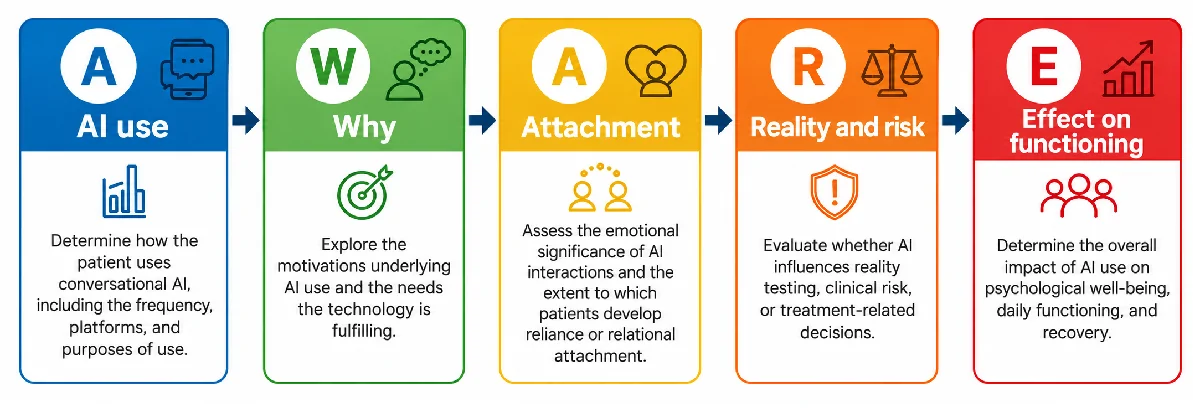
The AWARE (AI use, why, attachment, reality and risk, and effect on functioning) framework: a structured approach to assessing patients’ use of conversational AI.

### AI Use

#### Educational Objective

The educational objective for the *AI use* domain is to determine the patient’s exposure to conversational AI, including which platforms they use, how frequently and in what contexts they engage with them, the modalities through which they interact, and whether sensitive personal, health-related, or third-party information is shared.

#### Why It Matters

The nature of information shared with conversational AI may also be clinically relevant even when the pattern of use is otherwise adaptive. Patients may enter sensitive psychiatric symptoms, trauma histories, medication information, personal identifiers, medical documents, images, or information concerning family members and other third parties. Data handling, retention, secondary use, and privacy protections vary across platforms and may not be fully understood by users. A clinical AI history can therefore include a brief exploration of sensitive data disclosure without assuming that such disclosure reflects problematic AI use.

#### Suggested Questions

Questions suggested for the *AI use* domain are as follows:

- What types of personal or health information do you share with AI?
- Have you ever uploaded medical records, reports, images, or other health documents?
- Do your conversations ever include identifiable or sensitive information about other people?
- How do you decide what information you are comfortable sharing with an AI system?

Clinicians should characterize frequency of use, duration, and intensity. Occasional educational use may have little clinical significance, whereas prolonged daily interactions or exclusive reliance on AI for emotional support may warrant further exploration. Exposure itself should not be viewed as problematic but rather as contextual information that guides subsequent assessment.

### Why

#### Educational Objective

The educational objective for the *why* domain is to understand the motivations underlying AI use.

#### Why It Matters

The reason patients engage with AI is often more clinically informative than the amount of use itself. Similar patterns of use may have very different clinical implications depending on whether AI is being used for education, productivity, emotional support, companionship, or coping with psychological distress. Motivation helps clinicians understand the role AI occupies in relation to the patient’s broader psychological and social needs.

#### Suggested Questions

Questions suggested for the *why* domain are as follows:

- What do you find most helpful about AI?
- Can you tell me about the situations in which you choose to talk with AI or another person?
- What role, if any, does AI play when you are dealing with stress or difficult emotions?
- What needs does AI meet that other resources do not?

Clinicians should differentiate between motivations such as education, work productivity, creativity, health information, therapy or emotional support, coping, and companionship. Educational and productivity uses generally require little further exploration. Uses centered on emotional regulation, loneliness, reassurance seeking, companionship, or interpersonal needs may warrant further contextual exploration, particularly when they are associated with distress, unwanted displacement of other supports, or functional consequences.

### Attachment

#### Educational Objective

The educational objective for the *attachment* domain is to assess the emotional relationship between the patient and conversational AI.

#### Why It Matters

One of the defining characteristics of conversational AI is its capacity to support interactions that users may experience as relationally meaningful. Several distinct constructs may help characterize these interactions, including perceived therapeutic alliance, anthropomorphism, trust, companionship, parasocial interaction, emotional reliance, and attachment-related experiences. Although these constructs may overlap, they describe different aspects of patient-AI interaction and should not be treated as equivalent. Clinically, the purpose of this domain is therefore not to classify a patient along a single attachment trajectory, but to understand the emotional and relational significance that AI has acquired for that individual.

Although AI systems do not possess genuine emotions or reciprocal relationships, patients may nevertheless experience trust, comfort, companionship, or emotional security during repeated interactions.

#### Suggested Questions

Questions suggested for the *attachment* domain are as follows:

- How would you describe your relationship with AI?
- What is it like when you are unable to use AI?
- How would you describe your experience of being understood when interacting with AI?
- Can you describe how you decide when to trust AI and when to trust other people?
- Are there situations where you had personal conversations with AI? What happened?

#### Clinical Interpretation

The relational significance of conversational AI should not be reduced to a single continuum or assumed to progress through fixed stages. Different phenomena may be clinically relevant, including perceived therapeutic alliance, anthropomorphism, trust, companionship, emotional reliance, and dependence. These constructs overlap conceptually but are not interchangeable and may occur independently. Clinicians should therefore explore the nature, intensity, and functional significance of the patient’s relationship with AI without assuming that preference for AI necessarily reflects pathology or predicts increasing dependence.

### Reality and Risk

#### Educational Objective

The educational objective for the *reality and risk* domain is to identify whether AI interactions raise concerns requiring more focused assessment of (1) reality appraisal and judgment or (2) established areas of psychiatric risk and safety.

#### Why It Matters

The domain of *reality and risk* combines 2 clinically related but conceptually distinct areas of inquiry. They are grouped for mnemonic and interviewing purposes rather than because they represent a single psychological construct. The first concerns whether AI-generated content has become involved in the patient’s appraisal of reality, beliefs, or important decisions. The second concerns whether AI interactions have influenced established areas of psychiatric safety, such as suicidal behavior, self-harm, violence, treatment decisions, or unsafe health behaviors. In either case, positive findings should prompt assessment using established clinical approaches rather than interpretation through AWARE alone.

#### Reality Appraisal and Judgment

Questions suggested for the *reality and risk* domain under reality appraisal and judgment are as follows:

- How, if at all, have your conversations with AI influenced the way you think about yourself?
- How, if at all, have your conversations with AI influenced important decisions in your life?
- How would you compare your experience of being understood by AI with your experience of being understood by other people?
- Can you tell me about any situations in which AI responded differently to your beliefs or experiences than other people did?
- Have you ever used AI to understand what is real and what is not?
- How do you decide whether the information or advice provided by AI is accurate or trustworthy?

Clinicians should explore whether AI has become incorporated into self-identity, fixed beliefs, grandiosity, persecutory ideas, and delusional systems. Most patients will answer negatively. However, when positive responses occur, they should be interpreted within the broader clinical context rather than attributed solely to AI.

#### AI Involvement in Risk and Safety

AI may also become relevant during assessment of suicidal ideation, self-harm, violence, treatment refusal, medication discontinuation, and unsafe health behaviors. It is to be noted that AWARE does not assess suicide, self-harm, or violence risk. Questions in this domain are intended only to determine whether conversational AI has entered the context of an established area of psychiatric risk, for example, whether a patient has discussed suicidal thoughts with AI, sought crisis support from it, or reported that AI influenced a safety-related decision. Any concern regarding suicide, self-harm, violence, or other acute risk requires assessment using established clinical approaches independent of AWARE.

Questions suggested for the *reality and risk* domain under AI involvement in risk and safety are as follows:

- Can you tell me about any conversations you have had with AI about thoughts of suicide or self-harm?
- Can you tell me about any times you turned to AI during a crisis and what happened?
- What role, if any, has AI played in decisions about your treatment or medications?
- How, if at all, has AI influenced your decisions about seeking professional help?

Positive responses should prompt standard psychiatric risk assessment. AI should be understood as 1 component of a patient’s environment that may either mitigate or exacerbate existing vulnerabilities rather than as an independent cause of harmful behavior.

### Effect on Functioning

#### Educational Objective

The educational objective for the *effect on functioning* domain is to evaluate the overall impact of AI use on the patient’s functioning and quality of life.

#### Why It Matters

The clinical significance of AI use depends on its effects on everyday functioning. Many patients may experience substantial benefits, whereas others may encounter adverse consequences. Assessment should therefore remain balanced and avoid assuming either benefit or harm.

#### Suggested Questions

Questions suggested for the *effect on functioning* domain are as follows:

- How, if at all, has using AI influenced your daily routine?
- How, if at all, has using AI influenced your work or school performance?
- How, if at all, has using AI influenced your relationships?
- How, if at all, has using AI changed your sleep?
- How, if at all, has using AI influenced your mental health treatment?

Clinicians should evaluate effects on sleep, occupation, education, relationships, daily functioning, and quality of life. Assessment should identify both positive and negative outcomes. AI may improve coping, learning, and access to support while simultaneously contributing to excessive use, interpersonal withdrawal, reassurance seeking, or reduced engagement with treatment. Clinical interpretation should therefore emphasize the overall balance of benefits and harms within the context of the individual patient’s circumstances.

The suggested AWARE interview guide is presented in Table 1.

**Table 1:** The AWARE (AI use, why, attachment, reality and risk, and effect on functioning) interview guide.

Domains	Why assess?	Example questions	Potential concerns
AI use	Characterize exposure, context, and information sharing	Which AI tools do you use? How often? What types of personal or health information do you share?	Sensitive data disclosure, sharing of third-party information, privacy concerns, or intensive or exclusive use
Why	Understand motivations	What do you use AI for? Why do you prefer it?	Emotional coping, reassurance seeking, or social replacement
Attachment	Evaluate emotional relationship	Do you feel understood by AI? Do you rely on it emotionally?	Emotional reliance, dependence, anthropomorphism, or substitution for human relationships
Reality and risk	Assess influence on beliefs and safety	Has AI changed your beliefs or treatment decisions? Have you discussed suicide or self-harm with AI?	Delusion reinforcement, unsafe advice, treatment refusal, or crisis management
Effect on functioning	Evaluate clinical significance	Has AI affected your sleep, work, relationships, or recovery?	Functional impairment, treatment refusal, social withdrawal, or meaningful improvements in coping and functioning

### Documentation

When AI use emerges as clinically relevant, documentation may briefly capture the pattern of use, its psychological significance, and any identified benefits, concerns, or implications for care. AWARE may provide learners with a structure for organizing such documentation without prescribing a standardized documentation requirement. Documentation should describe the pattern of AI use, its psychological significance, and any identified risks or benefits.

A statement such as “uses ChatGPT” provides little clinically useful information. A more useful example would be as follows: “Patient uses ChatGPT approximately 1–2 hours daily for emotional support and problem solving. Reports feeling understood by the chatbot but does not describe preferring AI over interpersonal relationships. Patient reports no AI-related changes in important beliefs or treatment decisions and denies using AI for support related to suicidal or self-harm thoughts. Reports improved coping with anxiety and no perceived impairment in occupational, social, or academic functioning.”

This useful documentation describes the reported role of AI in the patient’s life; it should not be interpreted as documenting the absence of suicide risk, impaired reality testing, or other psychopathology. When clinically indicated, these areas require separate assessment and documentation using established psychiatric methods.

## Educational Implementation

AWARE is intended primarily as an educational framework across the continuum of health professions education. Its educational objective is to help learners recognize when a patients’ use of conversational AI may be clinically relevant and provide a structured approach to exploring it. The framework does not imply that every patient requires comprehensive AI-related questioning; rather, learners should develop the judgment to determine when brief inquiry is sufficient and when more detailed exploration is warranted.

At the undergraduate level, AWARE may introduce AI history taking alongside established psychosocial components of the clinical interview. In postgraduate psychiatry training, it may support more advanced formulation by encouraging learners to consider how AI interacts with symptoms, coping, relationships, treatment engagement, and safety. The same principles may be adapted for psychologists, nurses, social workers, family physicians, and practicing clinicians according to professional scope and clinical context (Table 2).

**Table 2:** Educational applications of the AWARE (AI use, why, attachment, reality and risk, and effect on functioning) framework.

Learner groups	Educational goals	Teaching methods
Medical students	Introduce AI history taking	Lectures, case-based learning, and simulation
Psychiatry residents	Advanced formulation and risk assessment	Clinical supervision, objective structured clinical examinations, and workplace-based assessment
Psychologists	AI and therapeutic processes	Case discussion and reflective practice
Nurses	Routine mental health assessment	Simulation and bedside teaching
Social workers	Social context and support systems	Case-based learning and supervision
Family physicians	Screening and referral	CPD^a^ workshops and workplace learning
Practicing clinicians	Updating interviewing skills	CPD

^a^CPD: continuing professional development.

Educational strategies may include case-based learning, simulation, objective structured clinical examinations (OSCEs), clinical supervision, and workplace-based assessment. For example, standardized patients could describe AI use for emotional support, health-related decision-making, or during psychological crises, allowing learners to practice identifying when additional clinical assessment is warranted. In workplace settings, existing approaches, such as the mini–clinical evaluation exercise (mini-CEX) or case-based discussion, could incorporate the ability to obtain an AI history, interpret its relevance, and document findings appropriately. These applications concern the teaching and assessment of interviewing skills and should not be interpreted as evidence that AWARE itself has been clinically validated.

Because conversational technologies continue to evolve, AWARE is intended as a flexible educational scaffold rather than a fixed checklist or competency standard.

## Future Directions

AWARE should be regarded as an educational interviewing framework rather than a diagnostic or psychometric instrument. It has not undergone formal development or validation, and its 5 domains should therefore be considered provisional. Initial research should establish content validity through structured input from clinicians, patients, and other stakeholders and determine whether the domains adequately capture clinically relevant patient-AI interactions. Studies should subsequently evaluate feasibility, acceptability, educational utility, and whether AWARE improves the completeness or clinical relevance of AI-related history taking.

Only if these initial evaluations are supportive should broader implementation or development of a standardized assessment instrument be considered. Future work should also examine cross-cultural applicability and whether emerging issues, including privacy, accessibility, and changing forms of patient-AI interaction, require modification of the framework.

## Conclusions

Conversational AI is rapidly becoming part of patients’ cognitive, emotional, and social lives. As individuals increasingly use AI for education, decision-making, emotional support, companionship, and mental health–related concerns, understanding these interactions is becoming an important component of contemporary psychiatric assessment. Despite this shift, clinicians currently receive little guidance on how to systematically explore patients’ AI use during routine clinical encounters.

The AWARE framework provides a structured educational approach to addressing this gap. By guiding clinicians through 5 domains (*AI use*, *why*, *attachment*, *reality and risk*, and *effect on functioning*), the framework supports comprehensive history taking, clinical reasoning, risk assessment, documentation, and learner education without assuming that AI use is inherently beneficial or harmful. Instead, it encourages individualized assessment that considers how conversational AI interacts with each patient’s symptoms, coping strategies, relationships, and overall functioning.

Although AWARE has not yet been formally validated and should not be considered a diagnostic, screening, or risk assessment instrument, it offers an educational starting point for preparing clinicians to recognize and explore clinically relevant patient-AI interactions. Its use in clinical practice should remain flexible and guided by presenting concerns rather than applied as a universal or comprehensive assessment. As conversational AI becomes increasingly embedded in everyday life, empirical evaluation will be needed to determine whether structured approaches such as AWARE improve education, interviewing, and patient care.

## Abbreviations

AWARE: AI use, why, attachment, reality and risk, and effect on functioning
CBT: cognitive behavioral therapy
LLM: large language model
mini-CEX: mini–clinical evaluation exercise
OSCE: objective structured clinical examination
